# Oscillatory Cortical Network Involved in Auditory Verbal Hallucinations in Schizophrenia

**DOI:** 10.1371/journal.pone.0041149

**Published:** 2012-07-23

**Authors:** Remko van Lutterveld, Arjan Hillebrand, Kelly M. J. Diederen, Kirstin Daalman, René S. Kahn, Cornelis J. Stam, Iris E. C. Sommer

**Affiliations:** 1 Department of Psychiatry, University Medical Center, Utrecht, the Netherlands and Rudolf Magnus Institute of Neuroscience, Utrecht, the Netherlands; 2 Department of Clinical Neurophysiology, VU University Medical Center, Amsterdam, the Netherlands; United Graduate School of Child Development, Osaka University, Japan

## Abstract

**Background:**

Auditory verbal hallucinations (AVH), a prominent symptom of schizophrenia, are often highly distressing for patients. Better understanding of the pathogenesis of hallucinations could increase therapeutic options. Magnetoencephalography (MEG) provides direct measures of neuronal activity and has an excellent temporal resolution, offering a unique opportunity to study AVH pathophysiology.

**Methods:**

Twelve patients (10 paranoid schizophrenia, 2 psychosis not otherwise specified) indicated the presence of AVH by button-press while lying in a MEG scanner. As a control condition, patients performed a self-paced button-press task. AVH-state and non-AVH state were contrasted in a region-of-interest (ROI) approach. In addition, the two seconds before AVH onset were contrasted with the two seconds after AVH onset to elucidate a possible triggering mechanism.

**Results:**

AVH correlated with a decrease in beta-band power in the left temporal cortex. A decrease in alpha-band power was observed in the right inferior frontal gyrus. AVH onset was related to a decrease in theta-band power in the right hippocampus.

**Conclusions:**

These results suggest that AVH are triggered by a short aberration in the theta band in a memory-related structure, followed by activity in language areas accompanying the experience of AVH itself.

## Introduction

Auditory verbal hallucinations (AVH) are one of the core symptoms of schizophrenia with approximately 70% of all schizophrenia patients presenting with these symptoms [1], [2]. AVH can be highly distressing and often lead to a disrupted social life [3]. Treatment for AVH would benefit from a detailed understanding of the pathophysiology of AVH. This, however, remains elusive.

An intuitive way to investigate AVH-related brain activity is to contrast the AVH state with the non-AVH state. Several studies have used this approach [4]–[14]. A recent meta-analysis implicated the bilateral insula, left middle and superior temporal gyrus, left hippocampus, left parahippocampal gyrus, left supramarginal gyrus, bilateral inferior frontal gyrus, and right internal globus pallidus in the experience of AVH [15].

Another strategy to study AVH is to identify brain regions that show changes in activity surrounding the start of hallucinations, as this may shed light on its triggering mechanism. Five functional magnetic resonance (fMRI) studies examined this topic [5], [16]–[19]. The three largest studies reported a decrease in activity in the parahippocampal gyrus preceding the experience of AVH, suggesting the involvement of memory processes in the genesis of AVH [5], [16], [17].

The vast majority of neuroimaging studies investigating AVH used fMRI. However, MRI scanner noise may interact with hallucination-related brain activity, and blood oxygen level-dependent (BOLD) activity is an indirect measure of neuronal activity. More importantly, the temporal resolution of fMRI is poor, confounding precise analysis of brain activity surrounding AVH onset. In contrast, magnetoencephalography (MEG) is a silent technique and directly measures postsynaptic neuronal activity. Moreover, it has a high temporal resolution, which makes it especially suitable to study short time-windows surrounding AVH onset. Thus far, MEG studies investigated AVH in one or a few (maximal 3) patients, reporting increases in power in the left superior temporal gyrus and left dorsolateral prefrontal gyrus (DLPFC) during AVH [7], [9], [10].

The first aim of the present study was to contrast the AVH and non-AVH state using MEG in a larger sample, enabling the use of group-level statistics in a region-of-interest (ROI) based design. The second aim was to investigate the neuronal correlates of AVH onset using MEG in a ROI based design.

## Methods

### Ethics statement

All patients gave their written informed consent and the study was approved by the ethics committees of the University Medical Center in Utrecht and the VU University Medical Center in Amsterdam.

### Subjects

Twenty schizophrenia-spectrum patients experiencing frequent auditory verbal hallucinations (AVH) were recruited at the University Medical Center in Utrecht, the Netherlands. Patients were diagnosed using the Comprehensive Assessment of Symptoms and History (CASH) interview [20] according to DSM-IV criteria by an independent psychiatrist. Eight out of twenty patients were excluded from analysis (3 patients experienced continuous AVH during recording, 3 patients did not experience AVH during recording, 1 experimental session was aborted because of anxiety of the patient, and we encountered technical difficulties during data-acquisition with 1 patient). Age and clinical characteristics for the 12 included patients (8 male, 4 female) are presented in table 1.

**Table 1:** Patient characteristics.

Subject	Sex	Age (years)	Age of onset AVH (years)	Handedness	Psychiatric diagnosis	Antipsychotic medication/day
A	M	41	21	Right	paranoid schizophrenia	clozapine 300 mg and risperidone 3 mg
B	M	36	34	Right	paranoid schizophrenia	no antipsychotic medication
C	F	52	19	Right	psychosis NOS; borderline personality disorder	clozapine 600 mg
D	M	39	19	Right	paranoid schizophrenia	chlorprothixene 200 mg
E	M	26	25	Left	paranoid schizophrenia	flupenthixol 6 mg
F	F	57	8	Right	paranoid schizophrenia	no antipsychotic medication
G	M	36	23	Right	paranoid schizophrenia	clozapine 400 mg
H	M	41	6	Right	paranoid schizophrenia	clozapine 300 mg
I	M	62	30	Right	psychosis NOS	no antipsychotic medication
J	F	57	9	Right	paranoid schizophrenia	quetiapine 600 mg
K	M	35	30	Right	paranoid schizophrenia	no antipsychotic medication
L	F	42	26	Right	paranoid schizophrenia	olanzapine 10 mg

AVH = auditory verbal hallucinations, M = male, F = female, NOS = not otherwise specified.

### Data acquisition

Brain activity was recorded at the VU University Medical Center using a 151-channel whole-head neuromagnetometer (CTF Systems Inc., Port Coquitlam, BC, Canada) in a magnetically shielded room (Vacuumschmelze GmbH, Hanau, Germany). Sampling rate was 625 Hz and the recording bandpass was 0 to 200 Hz. A third-order software gradient was applied for online noise cancellation [21]. At the beginning and end of each recording, the head position relative to the coordinate system of the helmet was recorded by leading small alternating currents through three head position coils attached to the subject's nasion and left and right pre-auricular points. Head position changes during the recording up to 1.0 cm were accepted. The three fiducial points were photographed for each participant for the purpose of co-registration of the MEG data to structural MRI scans.

During the MEG recording, patients lay in supine position and were instructed to close their eyes and move as little as possible. Subjects indicated the onset and offset of AVH by button-press. That is, subjects briefly pressed a button in the left hand at the start of AVH and briefly pressed a button in the right hand at the end of AVH. Neuromagnetic brain activity was recorded as continuous datasets of 1800 s duration. Due to practical considerations, the experiment was stopped early for 5 patients, resulting in datasets of 600, 1040, 1260, 1270 and 1770 seconds. To infer brain activation related to the button-presses, patients also performed a self-paced button-press task for 10 minutes, in which subjects alternatingly pressed the left and right buttons approximately every 10 seconds. Patients were specifically instructed not to relate the button-presses to the presence of AVH. Proper execution of this instruction was verbally verified after the experiment. Arousal levels were monitored online and subjects were prompted when arousal levels dropped; these data segments were excluded from the analysis.

To facilitate source localization of the MEG data, high-resolution magnetic resonance imaging (MRI) scans were obtained with a 3 Tesla MRI scanner (Philips Medical Systems, Best, the Netherlands) (TR/TE: 9.86/4.6 ms, 1 mm^3^ voxels, flip angle 8°). Co-registration of the MEG and MRI data was achieved by selecting the location of the nasion and pre-auriculars on the anatomical MRI. The fiducials were subsequently displayed on a 3D display of the head surface, as obtained from the segmented MRI, and visually compared with the location of the fiducials in the digital photographs taken during the MEG experiment. The location of the fiducials in the MRI was adjusted if there was a mismatch between the location of the fiducials on the MRI surface and the photographs. Severity of AVH during scanning was assessed directly after data acquisition using the Auditory Hallucinations Rating Scale (AHRS). This questionnaire assesses multiple characteristics of AVH such as the frequency of occurrence, loudness of the voices, length of AVH, and influence and discomfort of AVH as experienced by the patient [22]. In addition to the above, the Positive and Negative Syndrome Scale (PANSS) was used to assess clinical symptomatology [23].

### Data selection

Artifact-free segments were selected by visual inspection by two experienced MEG investigators (RvL and AH). In the present study, the neural correlates of AVH were investigated by contrasting the AVH state versus the non-AVH state. In addition, the neural correlates of AVH onset were investigated by contrasting the first 2 seconds of the AVH state versus the 2 seconds preceding AVH onset. Data selection procedures were different for both analyses:

- For the AVH-state vs. non-AVH state analysis, duration and number of selected time segments were identical for the hallucinatory and non-hallucinatory states within each subject, but these parameters were dissimilar across subjects in order to maximize the total amount of useful data for each participant. As the selection of longer segments led to fewer usable segments (because of large intra-individual heterogeneity in AVH and non-AVH duration and a higher chance of artifacts being present), maximization of data was achieved by selecting the segment length for which the product of number of segments and segment length was largest. Segments started at the button-presses' offset. To verify the effect of the button-presses, 5 s segments were selected from the control experiment, with segments starting at the left and right button-presses' offset. The number of segments in the control and AVH analysis was individually matched. Individual data regarding selected segments are provided in Table S1.
- For the onset of AVH analysis, 2 seconds prior and 2 seconds following AVH onset were selected. This timeframe was chosen because of practical considerations; selection of longer timeframes would result in a sharp drop in useful segments because of the large heterogeneity in AVH and non-AVH duration, presence of artifacts, and decreased matching possibilities with the control experiment. Individual data regarding selected segments are provided in Table S1. As a control condition, the same analyses were performed for the control experiment, with artifact-free segments of 2 seconds surrounding the left button-press serving as a control condition for the AVH onset analysis. The number of selected segments in the control analysis was matched individually to the AVH onset analysis, and duration of the incorporated button-press was matched at the group-level.

### Data analysis

Electromagnetic source analysis was conducted with the Synthetic Aperture Magnetometry (SAM) beamformer algorithm as implemented in the CTF software [24]. With this approach, an optimal spatial filter is constructed for each location in the brain individually using the entire array of MEG sensors. Estimates of neuronal activity in the target locations can then be obtained by projecting sensor power through the filter. These estimated timeseries have the same millisecond temporal resolution as the original MEG recording [25] and are usually referred to as *virtual electrode* signals [26]. Differential images of source power (pseudo-*t* maps) can then be constructed by contrasting the neuronal power in each location for two conditions (divided by a projection of the estimated sensor noise [27]. In this experiment, beamformer images were constructed on a 5 mm^3^ grid throughout the whole brain. Four separate frequency bands were selected for analysis (delta 0.5–4 Hz, theta 4–8 Hz, alpha 8–13 Hz, beta 13–30 Hz. The gamma frequency band (30–48 Hz) was not included as high-frequency bands may be contaminated by muscle artefacts [28].

The individual participants' beamformer images were spatially normalized to a template brain and then averaged across participants, following the procedure described in Singh et al [29]. As a nonparametric approach was most appropriate given the small sample size, statistical analysis was performed using a nonparametric permutation test (SnPM) [30], using all possible permutations. The family-wise error (FWE) correction threshold was set at P = 0.05, combined with an extent threshold of 6 contiguous voxels [31]–[33]. For the hypothesis-driven focus on specific areas, the analysis was conducted on anatomically defined ROIs, based on previous literature:

- For the AVH state versus non-AVH state analysis, ROIs were selected based on a recent neuroimaging meta-analysis that implicated the bilateral insula, left middle and left superior temporal gyri, left hippocampus and left parahippocampal gyrus, left supramarginal gyrus, bilateral inferior frontal gyrus, and right globus pallidus in the experience of AVH [15]. To increase sensitivity of the statistical tests, motor areas were not included in the mask, as any potential activation in these regions is likely to be related to the button-presses.
- For the AVH onset analysis, ROIs included the bilateral hippocampus and parahippocampal gyrus [5], [16], [17]. Recent studies have demonstrated the efficacy of MEG in detecting and localizing hippocampal activity [34]–[37]. In order to explore whether findings in this analysis are prolonged or primarily occurring in the small-time-frame surrounding AVH onset, this reduced mask was also applied to the AVH state versus non-AVH state data. Time-frequency plots were calculated at peak loci for each individual using a Morlet wavelet transform. These peak loci were located in close proximity to the peaks observed in the group analysis.

To elucidate potential associations between symptomatology and changes in neuronal activity, in a secondary analysis correlations between changes in delta, theta, alpha and beta-band activity in analysis i) and analysis ii) and scores on the AHRS and the positive symptom scale of the PANSS were investigated. Detailed information regarding this analysis is provided in Text S1. ROIs were defined using the WFU Pickatlas software tool (version 2.4, Wake Forest University, Winston-Salem, NC, USA; http://fmri.wfubmc.edu/cms/software) and the AAL library [38], and subsequently resliced to the coordinate system of the spatially normalized MEG datasets. To be able to assess cortical activity at the boundaries of the masks, both masks were inflated by 2 mm. Anatomical labelling of significant activation clusters was performed with the WFU Pickatlas using the AAL library, and MRIcron (http://www.sph.sc.edu/comd/rorden/mricron/) was used for visualization of group activation maps.

## Results

### Behavioral

Patients had an average of 35 AVH per 30 minutes during the experiment (SD 25; range 12–88). Average duration of AVH was 27 s (SD 18; range 6–66) and average duration of the non-AVH periods was 40 s (SD 30; range 2–108). Mean total AHRS score was 26 (SD 6; range 19–41). Mean total score on the positive subscale of the PANSS was 16 (SD 3; range 11–22), mean total score on the negative subscale was 15 (SD 5; range 10–26), and mean total score on the general psychopathology subscale was 28 (SD 7; range 21–42).

### AVH versus non-AVH state analysis

**Figure 1:**
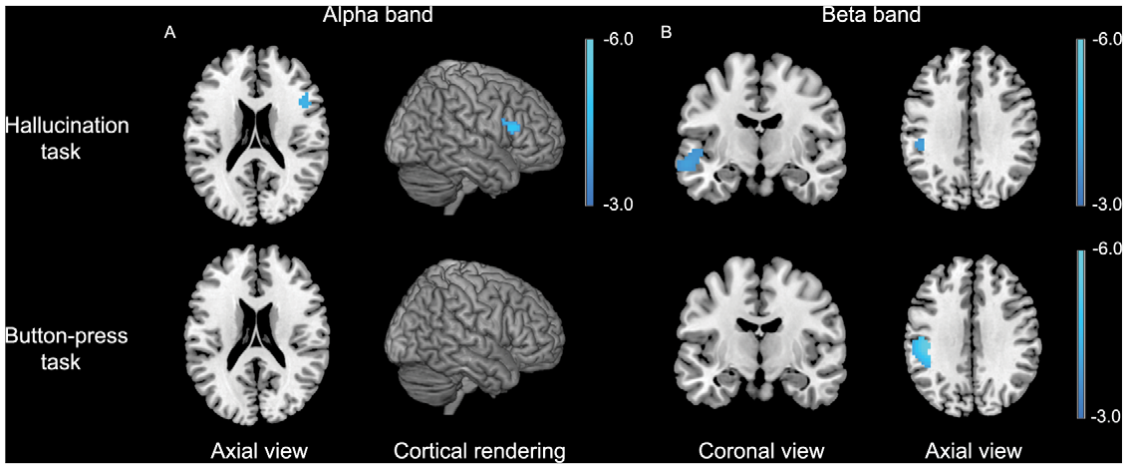
Results for the AVH versus non-AVH analysis for the alpha and beta frequency bands. Data are superimposed on a template brain and are presented in neurological convention (right is right). P<0.05, FWE corrected. The color bar indicates t-values.

MEG imaging revealed a statistically significant relative decrease in alpha-band neuronal power in the right inferior frontal gyrus, while no changes in alpha-band power were found in the control experiment (fig. 1a). For the beta band, significant relative decreases in power were observed in the left middle temporal gyrus extending into the left superior gyrus and in the left supramarginal gyrus. Changes in beta-band power in the latter region was also found in the control experiment (fig. 1b). Detailed information about the coordinates of local maxima and corresponding cluster sizes is shown in table 2.

**Table 2:** Significantly activated voxels and locations of local maxima during auditory verbal hallucinations in the group hallucination task and button-press task.

Task	Frequency band	Increase/decrease power	Lobe	Area	Montreal Neurological Institute Coordinates (x, y, z)	t	Cluster size
Hallucination task	Delta	-					
	Theta	-					
	Alpha	Decrease	Right frontal	Inferior frontal gyrus	48 21 15	4.88	36
	Beta	Decrease	Left temporal	Superior temporal gyrus/Middle temporal gyrus	−60 −12 −12	4.04	48
		Decrease	Left parietal	Supramarginal gyrus	−48 −33 33	4.00	12
Button-press task	Delta	-					
	Theta	-					
	Alpha	-					
	Beta	Decrease	Left parietal	Supramarginal gyrus/Postcentral gyrus	−42 −24 33	5.76	229

### AVH onset analysis

**Figure 2:**
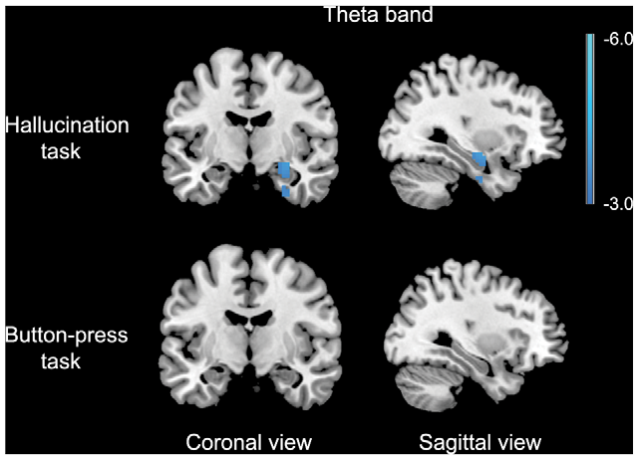
Results for the AVH onset analysis superimposed on a template brain. Data are presented in neurological convention (right is right). P<0.05, FWE corrected. The color bar indicates t-values.

A significant relative decrease in theta-band power was found in the right hippocampus extending into the amygdala in the 2 seconds following AVH onset versus the 2 seconds preceding AVH onset. For the self-paced button-press task, no change in theta-band power was found (fig. 2), while for the other frequency-bands no changes in power were observed in both experiments. Detailed information about the coordinates of local maxima and corresponding cluster sizes are shown in table 3, and time-frequency representations of two representative subjects are shown in Figure S1. Application of the AVH-onset mask to the AVH state versus non-AVH state data yielded no significant results in any frequency band.

**Table 3:** Significantly activated voxels and locations of local maxima in the group hallucination onset analysis for the hallucination task and the control task.

Task	Frequency band	Increase/decrease power	Lobe	Area	Montreal Neurological Institute Coordinates (x, y, z)	t	Cluster size
Hallucination task	Delta	-					
	Theta	Decrease	Right temporal	Hippocampus/Amygdala	30 −9 −12	3.93	28
	Alpha	-					
	Beta	-					
Button-press task	Delta	-					
	Theta	-					
	Alpha	-					
	Beta	-					

### Associations between clinical symptoms and changes in neuronal activity

No significant correlations were found between total AHRS scores and changes in neuronal activity related to either the experience of AVH or the onset of AVH. Sum scores of the positive symptom scale of the PANSS correlated positively with AVH-related changes in beta band power in the left inferior frontal gyrus (k = 27; MNI coordinates −18 21 −18), indicating that increased levels of positive symptoms were related to increased changes in beta-band neuronal power during the occurrence of AVH in this structure. No significant correlations were found between sum scores on the positive symptom scale of the PANSS for the AVH onset analysis.

## Discussion

This is the first study to utilize the excellent temporal resolution of MEG to investigate brain activity related to AVH onset. It is also the first study investigating the neural correlates of AVH employing MEG in a substantial sample (n = 12), enabling group-wise analysis.

AVH were associated with a decrease in alpha-band power in the right inferior frontal gyrus and with a decrease in beta-band power in the left middle and superior temporal gyrus. The motor control task did not result in any changes in neuronal power in these areas. AVH onset was accompanied by a decrease in theta-band power in the right hippocampus while in the control task no change in theta-band power was observed in this brain structure.

### AVH state versus non-AVH state analysis

AVH-related changes in the left temporal cortex, where we observed a decrease in beta-band power, are usually interpreted as auditory processing related to the perception of voices [6]. It has been suggested that beta oscillations are involved in underscoring a stimulus as novel or salient [39], [40]. The observed change in activity in this frequency band during the experience of AVH is as such in line with the fact that AVH are often of high emotional content (frightening) and therefore of high salience. An alternative explanation linking the decrease in beta-band power to AVH may be that the process of corollary discharge (i.e. a signal originating in frontal speech production areas that is sent to auditory perception areas to indicate that forthcoming thought is self-generated) is disturbed through the decrease in oscillatory power in this frequency band. For the observed decrease in alpha-band power in the right inferior frontal gyrus several explanations are possible, including processing of linguistic emotional information, detection of salient events and dysfunctional inhibition processes [41]–[43].

The present findings are partially consistent with those of previous studies. Functional MRI studies have implicated changes in activity in the left middle and left superior temporal cortex and right inferior frontal gyrus during AVH [5], [13], [15], [44]. Our findings are also partially in line with previous MEG studies, reporting increases in theta-band and beta-band power in the left superior temporal gyrus (STG) [7], [10], and an increase in beta-band activity in the left STG extending into the left dorsolateral prefrontal cortex (DLPFC) during AVH [9]. In the current study, the left STG was associated with a decrease instead of an increase in beta-band power during AVH. The previously published MEG studies reported single cases [7], [10] or three cases analyzed individually [9]. Single subject analyses yield more variable and less reliable results than group-wise analysis, providing a possible explanation for the divergent findings [45]. An EEG study also implicated the left STG, albeit in the alpha band [46]. The divergent findings in frequency bands between EEG and MEG may be explained by differences in methodologies. EEG sensors pick up electrical activity related to neuronal activity and these signals are smeared out by the skull. MEG measures magnetic activity, which is not substantially affected by the skull.

### AVH onset analysis

We observed a decrease in theta-band power in the hippocampus during AVH onset. Animal research of the hippocampus has revealed some of the functions of theta-band oscillations in memory retrieval. Cell recordings in the hippocampus in rat models have provided support that theta and gamma oscillations together form a code for neural representations of memorized items [47], [48]. Each cycle of the fast gamma rhythm is generated by a different neural ensemble, and represents a single item in memory [49], [50]. The gamma cycles are ordered by much slower theta-waves, i.e. theta waves form the ‘punctuation’ of the gamma oscillations and as such the items in memory they represent [49]. Moreover, the theta waves on which the gamma oscillations are superimposed coordinate the pattern of neuronal firing over larger brain areas [51]. The proposed function of theta oscillations would be to integrate different representations of perception, memory and association into one coherent concept, e.g. a congruent line of thoughts and experiences [49], [51]. The onset of a hallucination can be viewed as an interruption of this coherent line [51]. Hence, the change in theta rhythm observed in the hippocampus and parahippocampal gyrus at the onset of hallucinations may reflect the cessation of a coherent line of thoughts and perception, and perhaps the transition from a state of consciousness that is focused around perception of information from the sense organs to a state that relies to a higher degree on internal representations collected from memory.

In the present study no changes in hippocampal theta-band activity were found in the AVH state versus the non-AVH state, yet a transient change in theta-band activity surrounding AVH onset was found, suggesting that a temporary disturbance in theta-band activity may underlie the genesis of hallucinations through a short aberration in theta-band mediated integration of different representations. The present findings of changes in neuronal power in the theta band in the hippocampus surrounding AVH onset are partially in line with previous fMRI studies, which reported changes in activity in the parahippocampal gyrus to precede the onset of AVH [5], [16], [17]. This finding is also consistent with a recent cognitive model that postulates that AVH arise from dysfunctioning in memory processes and top-down inhibitory processing, in which the latter may be mediated by the right inferior frontal gyrus [42], [52]–[54].

As AVH often consist of emotional content, future research could focus on elucidating the relationship between emotional valence of AVH and neuronal activity related to the symptom [55].

### Limitations

A limitation of this study is that AVH were assessed by self-report, rendering the precision of AVH onset and offset subject to the patients' accuracy. Furthermore, we used a self-paced button-press paradigm as a control task. Although this paradigm is often used to investigate the effect of button-presses in a hallucination task [13], [17], the process of attention may not be similar in both tasks, as patients are required to press the buttons to indicate the presence of hallucinations in the hallucination task (‘externally triggered’) and the control task is self-paced (‘internally triggered’). As such, we recommend that future studies incorporate an additional non-self-paced control task, e.g. indicating the presence of externally presented auditory stimuli. Another limitation is that the presence of AVH during the control task may have influenced results. This is however not likely, as the self-paced button-presses were not related to the presence of AVH, leading to the presence of AVH in both parts of the contrast, and resulting in presumably no or minor impact on the analysis. Finally, most of the patients in the current study were on antipsychotic medication. This may have influenced results, as antipsychotic agents have been reported to affect theta-band activity [56].

To summarize, this is the first study focusing on the small time-frame surrounding AVH onset which could identify the triggering mechanism of hallucinations. The onset of AVH was accompanied by changes in theta-band power in the right hippocampus. Furthermore, AVH were associated with a decrease in alpha-band power in the right inferior frontal gyrus and with decreases in beta-band power in the left middle and superior temporal gyri, which are regions generally implicated in auditory and language processes. These results suggest that AVH are triggered by a short aberration in the theta band in the hippocampus, followed by activity in auditory areas accompanying the experience of hearing voices. We speculate that these aberrations may disturb the coherency of thoughts and perception such that there is an increased focus on internal representations collected from memory. New treatment options, such as deep brain stimulation, may target hippocampal structures in order to restore their normal function and impede the onset of AVH.

## Supporting Information

Figure S1 **Time-frequency representation (TFR) plots of source peak activity in the right hippocampus surrounding AVH onset in two representative subjects.** The frequency band in which a significant difference was observed in this brain region (group analysis) is indicated by the black box. The vertical line indicates the onset of hallucinations. TF: time-frequency. AVH: auditory verbal hallucinations.
(TIF)

Table S1 **Length and number of selected segments for the auditory verbal hallucination (AVH) analysis and the AVH onset analysis per subject.**
(DOC)

Text S1 **Supporting text.**
(DOC)
