# Supplementary figures and images for: Oscillatory Cortical Network Involved in Auditory Verbal Hallucinations in Schizophrenia
Source: PLoS One. 2012 Jul 23;7(7):e41149. doi: 10.1371/journal.pone.0041149 (PMC3402538; doi:10.1371/journal.pone.0041149)

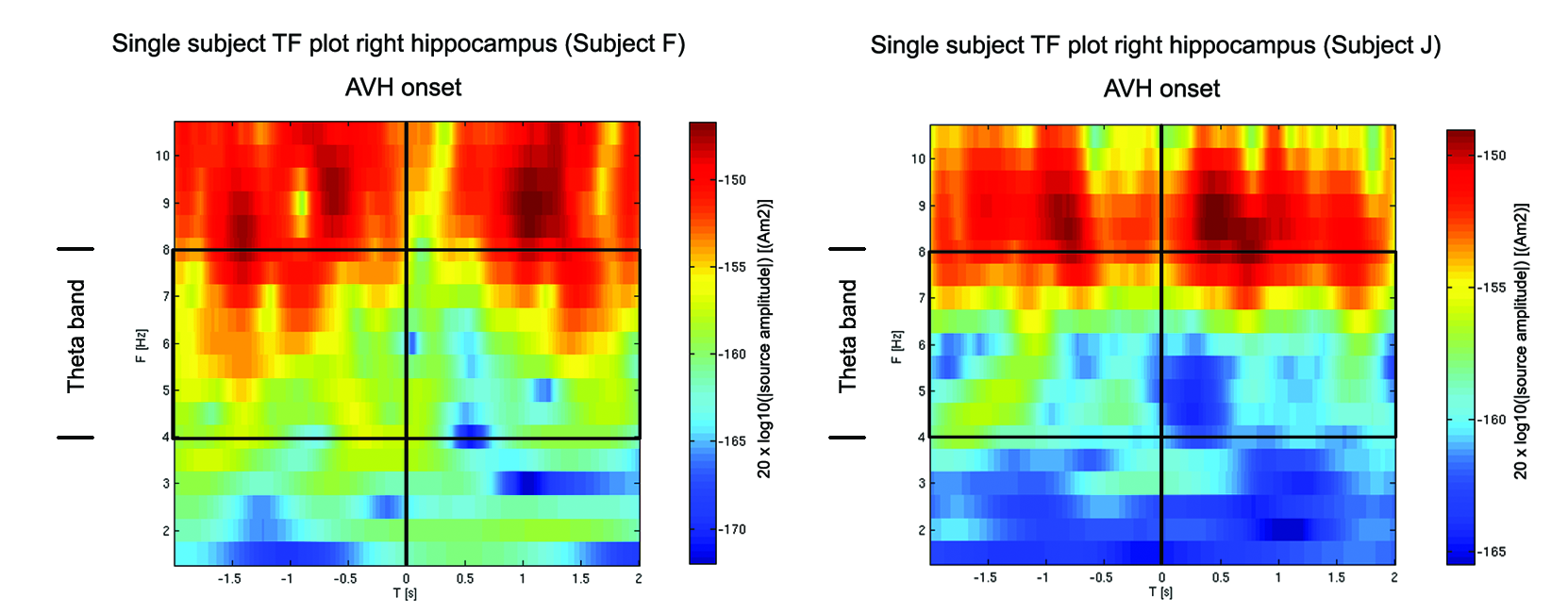

Supplement: Figure S1 — Time-frequency representation (TFR) plots of source peak activity in the right hippocampus surrounding AVH onset in two representative subjects. The frequency band in which a significant difference was observed in this brain region (group analysis) is indicated by the black box. The vertical line indicates the onset of hallucinations. TF: time-frequency. AVH: auditory verbal hallucinations. (TIF) [file pone.0041149.s001.tif]
