# Supplementary material for: Oscillatory Cortical Network Involved in Auditory Verbal Hallucinations in Schizophrenia
Source: PLoS One. 2012 Jul 23;7(7):e41149. doi: 10.1371/journal.pone.0041149 (PMC3402538; doi:10.1371/journal.pone.0041149)
Supplement: Table S1 — Length and number of selected segments for the auditory verbal hallucination (AVH) analysis and the AVH onset analysis per subject. (DOC) [file pone.0041149.s002.doc]

|  | Subject | A | B | C | D | E | F | G | H | I | J | K | L |
| --- | --- | --- | --- | --- | --- | --- | --- | --- | --- | --- | --- | --- | --- |
| AVH and non-AVH segments | Length (s) | 8.1 | 15.1 | 25.2 | 14.1 | 4.0 | 16.0 | 10.8 | 6.2 | 7.0 | 5.0 | 5.0 | 5.8 |
|  | Number | 13 | 8 | 5 | 9 | 29 | 6 | 4 | 22 | 13 | 5 | 6 | 12 |
|  | Total (s) | 105.3 | 120.8 | 126.0 | 126.9 | 116.0 | 96.0 | 43.2 | 136.4 | 91.0 | 25.0 | 30.0 | 69.6 |
| Before and after AVH onset segments | Length (s) | 2.0 | 2.0 | 2.0 | 2.0 | 2.0 | 2.0 | 2.0 | 2.0 | 2.0 | 2.0 | 2.0 | 2.0 |
|  | Number | 20 | 12 | 3 | 14 | 26 | 4 | 5 | 25 | 17 | 9 | 7 | 16 |
|  | Total (s) | 40 | 24 | 6 | 28 | 52 | 8 | 10 | 50 | 34 | 18 | 14 | 32 |
