# Supplementary material for: Oscillatory Cortical Network Involved in Auditory Verbal Hallucinations in Schizophrenia
Source: PLoS One. 2012 Jul 23;7(7):e41149. doi: 10.1371/journal.pone.0041149 (PMC3402538; doi:10.1371/journal.pone.0041149)
Supplement: Text S1 — Supporting text. (DOC) [file pone.0041149.s003.doc]

To elucidate potential associations between symptomatology and changes in neuronal activity, correlations between total AHRS scores and total PANSS positive subscale scores on one hand and changes in delta, theta, alpha and beta-band activity on the other hand were investigated.

1. Correlations between total AHRS scores and changes in neuronal activity in the AVH state versus non-AVH state analysis were investigated for each ROI separately (10 ROIs: the left insula, right insula, left middle and left superior temporal gyri, left hippocampus and left parahippocampal gyrus, left supramarginal gyrus, left inferior frontal gyrus, right inferior frontal gyrus, and right globus pallidus.
2. Correlations between total AHRS scores and changes in neuronal activity during AVH onset were investigated for each ROI separately (4 ROIs: the left parahippocampal gyrus, right parahippocampal gyrus, left hippocampus and right hippocampus).

Non-parametric, multi-subject simple regression analyses were carried out to investigate whether total AHRS scores were correlated with changes in neuronal activity in any of the predefined ROIs using SnPM (5000 permutations). To correct for multiple comparisons, the family-wise error (FWE) correction threshold was set at P = 0.0017 (0.05 divided by the total number of tests), combined with an extent threshold of 6 contiguous voxels.
